# The Holliday junction resolvase RecU is required for chromosome segregation and DNA damage repair in *Staphylococcus aureus*

**DOI:** 10.1186/1471-2180-13-18

**Published:** 2013-01-28

**Authors:** Ana R Pereira, Patricia Reed, Helena Veiga, Mariana G Pinho

**Affiliations:** 1 Laboratory of Bacterial Cell Biology, Instituto de Tecnologia Química e Biológica, Universidade Nova de Lisboa, Av. da República, Oeiras 2780-157, Portugal

**Keywords:** *Staphylococcus aureus*, *recU*, Chromosome segregation, DNA repair

## Abstract

**Background:**

The *Staphylococcus aureus* RecU protein is homologous to a *Bacillus subtilis* Holliday junction resolvase. Interestingly, RecU is encoded in the same operon as PBP2, a penicillin-binding protein required for cell wall synthesis and essential for the full expression of resistance in Methicillin Resistant *S. aureus* strains. In this work we have studied the role of RecU in the clinical pathogen *S. aureus.*

**Results:**

Depletion of RecU in *S. aureus* results in the appearance of cells with compact nucleoids, septa formed over the DNA and anucleate cells. RecU-depleted cells also show increased septal recruitment of the DNA translocase SpoIIIE, presumably to resolve chromosome segregation defects. Additionally cells are more sensitive to DNA damaging agents such as mitomycin C or UV radiation. Expression of RecU from the ectopic chromosomal *spa* locus showed that co-expression of RecU and PBP2 was not necessary to ensure correct cell division, a process that requires tight coordination between chromosome segregation and septal cell wall synthesis.

**Conclusions:**

RecU is required for correct chromosome segregation and DNA damage repair in *S. aureus*. Co-expression of *recU* and *pbp2* from the same operon is not required for normal cell division.

## Background

All cells have to repair DNA lesions caused not only by DNA damaging agents but also under normal growth conditions. Chromosome replication is not a continuous process and a series of barriers such as tightly bound proteins, abnormal DNA structures and DNA damage can cause replication fork arrest, which is a major source of genome instability [1-3]. In order to surpass these obstacles, bacteria have developed mechanisms to grant faithful inheritance of genomic information. One example is the process of homologous recombination, required to re-establish stalled and collapsed replication forks and to repair double strand breaks (DSBs) [4,5]. DSB repair is initiated by recognition of the damaged DNA, followed by processing of its ends, leaving a 3’ overhanging strand. The RecA protein associates with these overhanging strands, strand invasion occurs and a Holliday junction is formed and extended unidirectionally by branch migrating proteins such as RuvAB [6]. Holliday junction resolvases, such as *Bacillus subtilis* RecU, have multiple roles during this process as they promote RecA-mediated strand invasion, associate with the branch migrating proteins and resolve the Holliday junction through DNA cleavage [7-9]. The replication fork can then be re-established, generating either crossover or non-crossover products [10,11]. Importantly, *B. subtilis* RecU biases homologous recombination towards non-crossover products, therefore avoiding the formation of dimeric chromosomes that cannot be segregated to daughter cells in the absence of a compensating recombination reaction [11].

In agreement with the role of RecU in homologous recombination and DNA damage repair, *B. subtilis recU* mutants show several chromosome segregation defects. These include nucleoids that are bisected by the division septa, abnormal nucleoid position and anucleate cells [11,12], as well as an increased susceptibility to DNA damaging agents such as mitomycin C (MMC), methyl methanesulfonate (MMS) and UV light [13,14].

Homologous recombination is involved in the transfer of DNA within and, occasionally, between species, which can lead to acquisition of new traits including increased virulence or antibiotic resistance [15,16]. It is therefore of particular relevance to study this process in clinical pathogens. In this work, we focus on *Staphylococcus aureus,* an important clinical pathogen responsible for high mortality rates in hospitals, mainly due to the presence of methicillin-resistant *S. aureus* (MRSA) strains [17,18]. The study of RecU in *S. aureus* is relevant not only because of its putative role in homologous recombination, but also because it is encoded by the same operon as PBP2. This penicillin-binding protein is required for cell wall synthesis and essential for the full expression of resistance in MRSA strains [19,20]. It is interesting that a protein involved in homologous recombination and a protein involved in cell wall synthesis, two biochemically independent processes, are part of the same operon. Importantly, this genetic organization is conserved in other gram-positive bacteria, such as *Streptococcus pneumoniae*[21] and *B. subtilis*[22].

During cell division, the processes of chromosome replication and septum synthesis have to be tightly coordinated to avoid the disastrous consequences of DNA guillotining by a septum forming over the DNA. Since PBP2 is required for septum synthesis and RecU is apparently involved in chromosome segregation, we wondered if the regulation of this operon could constitute a possible checkpoint for cell division coordination in *S. aureus*. Here we show that *recU* absence causes cell growth defects due to an inability of the mutant to repair damaged DNA and to properly segregate the chromosomes. We also show that co-expression of *recU* and *pbp2* from the same operon is not required for normal cell division.

## Methods

### Bacterial strains and growth conditions

All strains and plasmids used in this study are listed in Table 1 and primer sequences are listed in Table 2. *S. aureus* strains were grown in tryptic soy broth (TSB, Difco) or on tryptic soy agar (TSA, Difco) at 37°C with aeration. The medium was supplemented when required with appropriate antibiotics (erythromycin 10 μg/ml, chloramphenicol 10 μg/ml), with 5-bromo-4-chloro-3-indolyl β-D-galactopyranoside 100 μg/ml (X-Gal; BDH Prolabo) or with isopropyl-β-D-thiogalactopyranoside 0.5 mM (IPTG; VWR).

**Table 1:** Strains and plasmids used in this study

**Strain/Plasmid**	**Relevant characteristics**	**Source/ Reference**
*E. coli*		
DH5α	Cloning strain, *recA endA1 gyrA96 thi-1 hsdR17 supE44 relA1* ϕφ80 Δ*lacZ*ΔM15	Gibco-BRL
*S. aureus*		
NCTC8325-4	MSSA strain	R. Novick
BCBHV008	NCTC8325-4Δ*spa*::P_*spac*_-MCS-*lacI lacI*^mc^, Cm^r^	[23]
8325-4Δ*recU*	NCTC8325-4 *recU* mutant lacking initial 165 codons	This study
8325-4*recUspaL*	NCTC8325-4 Δ*spa*::P_*spac*_-*recU*-*lacI*	This study
BCBRP001	NCTC8325-4 Δ*recU* Δ*spa*::P_*spac*_-*recU*-*lacI*	This study
8325-4*recU*i	NCTC8325-4 Δ*recU* Δ*spa*::P_*spac*_-*recU*-*lacI lacI*^mc^, Cm^r^	This study
BCBHV017	BCBHV008 strain expressing *spoIIIE*-*yfp* from the native chromosomal locus, Cm^r^	This study
BCBRP002	8325-4*recU*i mutant strain expressing *spoIIIE*-*yfp*, Cm^r^	This study
Plasmids		
pMAD	*E. coli – S. aureus* shuttle vector with the *bgaB* gene encoding a ß-galactosidase and thermosensitive origin of replication for Gram-positive bacteria, Amp^r^ Ery^r^	[24]
pBCB13	pMAD derivative with P_*spac*_-*lacI* between up- and downstream regions of the *spa* gene, Amp^r^ Ery^r^	[25]
pMGPII	Plasmid encoding *lacI* gene; Cm^r^	[26]
pMAD*recU*KO	pMAD derivative used for deletion of the first 165 codons of *recU,* Amp^r^ Ery^r^	This study
pBCB13*recUspa*L	pBCB13 derivative containing P_*spac*_-*recU-lacI* Amp^r^ Ery^r^	This study
pMUTINYFPKan	Integrative vector for C-termini YFP fusions; Amp^r^, Kan^r^	[27]
pBCBHV007	pMUTINYFPKan containing *spoIIIE–yfp* Amp^r^, Kan^r^	This study
pMAD*spoIIIEyfp*	pMAD derivative containing 3’ end *spoIIIE* –linker-yfp, Amp^r^ Ery^r^	This study
pBCBHV008	pMAD derivative containing 3’ end *spoIIIE*–linker-*yfp*-3’ 64 bp and downstream region of *spoIIIE*, Amp^r^ Ery^r^	This study

*lacI*^mc^ – cells expressing multicopies of the *lacI* gene (encoded by pMGPII).

**Table 2:** Primers used in this study

**Primer name**	**Sequence (5’-3’)**
recUp1	ATCGAGATCTATGTACTTCAGGTGCGT
recUp2	TAGACTTTTTAAAATTTCACCACACAAGTTTGGTAG
recUp3	ACTTGTGTGGTGAAATTTTAAAAAGTCTATAAC
recUp4	ATCGGGATCCCAATGTTTTGACGTTC
recUp5	TGGTGTATTGTGTCTTTCG
recUp6	TTCCCACCATTATTACCG
recUp7	ATCTGCATGCTTAATTATGTTGGC
recUp8	ATACCCGGGTGTGTGGTGAAATTTATG
recUp9	TATGCTCGAGTCATACGCGGTCC
spoIIIEp1	GCTGCGGTACCGTCATAGCTATTTTAGTAGTTG
spoIIIEp2	GCTGCGGTACC**GGAGGCGCCGCAGGA**CACCTCGTCATTATTAAGATC
spoIIIEp3	TGAGGATCCGATGAAAAATTCCCGTCT
spoIIIEp4	TACTCCCCGGGTTACTTGTACAGCTCGTCC
spoIIIEp5	TACTCCCCGGGCGGTCCACAAAAAGGAAG
spoIIIEp6	TGCATTCCATGGGACATGCTGATCTTTGAATTTTGAAATTG

Underlined sequences correspond to the restriction site. Bold sequences correspond to the five codon linker.

### Construction of a RecU null mutant

**Figure 1:**
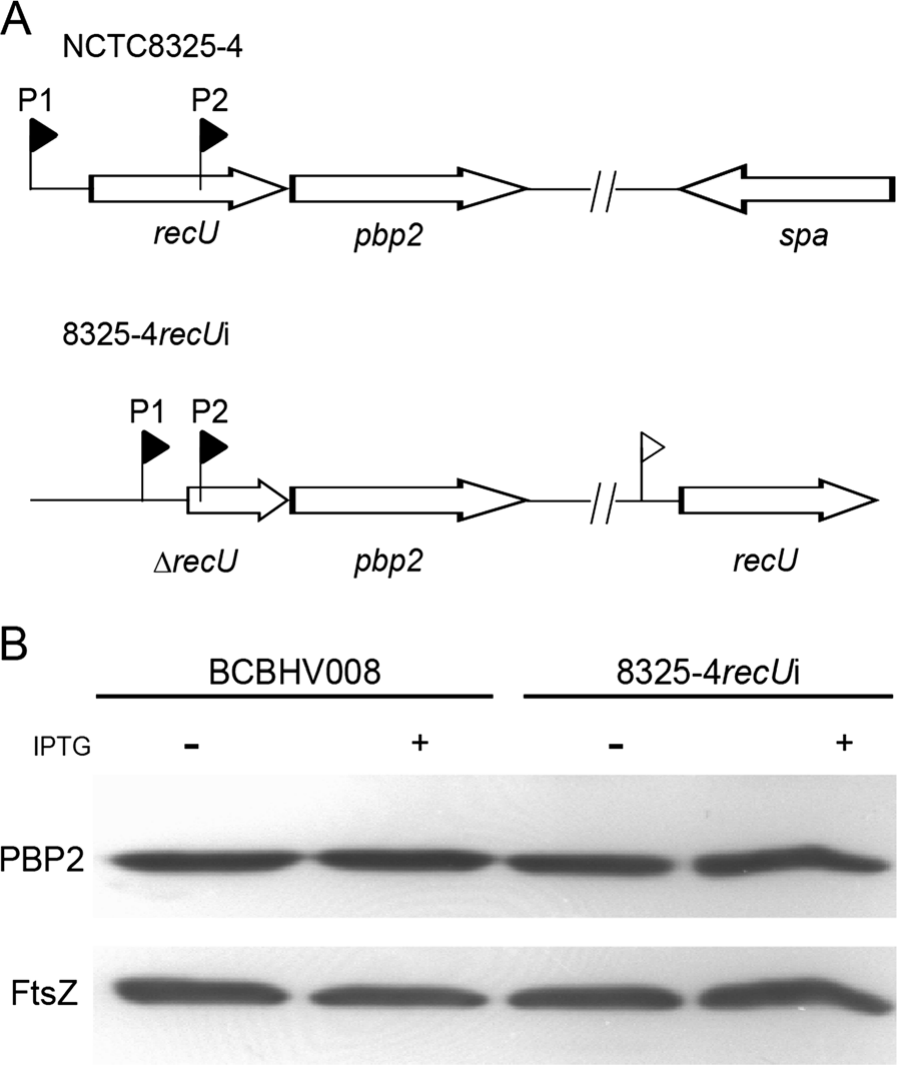
**RecU and PBP2 are encoded in the same operon. A** – Schematic representation of the *recU*-*pbp2* operon in the NCTC8325-4 wild-type strain (top) and the 8325-4*recU*i mutant strain (bottom) where the *recU* gene, including the RBS, was placed in the *spa* locus under the control of the IPTG inducible P_*spac*_ promoter (white flag). Subsequently, the first 165 codons of the native copy of *recU* were deleted*.* Black flags represent the promoters (P1 and P2) of the *recU*-*pbp2* operon. **B** – Western blot analysis of PBP2 levels in control strain BCBHV008 and *recU* inducible mutant 8325-4*recU*i grown in the presence or absence of IPTG showing that PBP2 levels were not affected by *recU* deletion. FtsZ was used as an internal control of total protein loaded.

To construct a *S. aureus recU* mutant lacking the initial 165 codons we amplified two 1 Kb DNA fragments, one containing the upstream region of *recU* up to its start codon (using primers recUp1 and recUp2), and the other containing the 3’end of *recU* including promoter P2 (see Figure 1A) [19] and the 5’ region of *pbp2* (using primers recUp3 and recUp4). The resulting PCR products were joined by overlap PCR using primers recUp1 and recUp4. The PCR product was digested with BamHI and BglII and cloned into the thermosensitive pMAD plasmid [24], resulting in plasmid pMAD*recU*KO. The insert was sequenced and the plasmid was electroporated into the transformable *S. aureus* strain RN4220 as previously described [28]. The plasmid was subsequently transduced to strain NCTC8325-4 using phage 80α [29] and insertion and excision of pMAD*recU*KO into the chromosome was performed as previously described [24]. Deletion of *recU* was confirmed by two different PCR reactions using the primers recUp5/recUp6 and recUp7/recUp6 and the resulting strain was named 8325-4Δ*recU*.

### Construction of a *recU* inducible mutant

To generate an inducible *recU* mutant, a full copy of the *recU* coding sequence was first placed in the *spa* locus under the control of the IPTG inducible-P_*spac*_ promoter using the pBCB13 plasmid [25]. The sequence encoding the first 165 amino acids of RecU was subsequently deleted from the native chromosomal locus using plasmid pMAD*recU*KO as described above. To clone *recU* into the *spa* locus, the entire *recU* coding sequence and the RBS was amplified by PCR using primers recUp8 and recUp9. The PCR product was digested with XmaI and XhoI restriction enzymes and cloned into pBCB13 generating the plasmid pBCB13*recUspaL*. The insert was sequenced, the plasmid was introduced into RN4220 by electroporation and subsequently transduced into NCTC8325-4. Integration and excision of the plasmid in the chromosome was performed as previously described [24] and the resulting strain, which contains two copies of *recU* in the chromosome, one in the native locus and another in the *spa* locus, was named 8325-4*recUspaL*. In order to delete *recU* from its normal locus in the background of strain 8325-4*recUspaL*, the pMAD*recU*KO plasmid was transduced into this strain and deletion of the *recU* gene was performed and verified as described in the previous paragraph, but in the presence of IPTG, resulting in the strain BCBRP001. In order to ensure tight regulation of the expression of *recU* from the P_*spac*_ promoter [30] we transduced the pMGPII plasmid, which encodes the *lacI* gene [26], into BCBRP001 and the resulting strain was named 8325-4*recU*i.

### SpoIIIE-YFP localization

To study SpoIIIE localization in BCBHV008 [23] and 8325-4*recU*i strains, derivatives of these strains expressing a C-terminal SpoIIIE-YFP fusion from its native locus were constructed. For that purpose, a DNA fragment encompassing a copy of the *spoIIIE* gene without its STOP codon and encoding a five amino acid linker was cloned, in frame with the *yfp* gene, in the pMUTINYFPKan plasmid [27]. This fragment was amplified from NCTC8325-4 genomic DNA using primers spoIIIEp1 and spoIIIEp2, digested with KpnI and cloned into pMUTINYFPKan, giving rise to pBCBHV007. The insert in pBCBHV007 was sequenced and this plasmid was used as a template to amplify a DNA fragment containing the 3’ end of the *spoIIIE* gene (1065 bp) connected to the linker and the *yfp* gene, using primers spoIIIEp3 and spoIIIEp4. This fragment was digested and cloned into the BamHI and XmaI restriction sites of the pMAD vector [24], generating plasmid pMAD*spoIIIEyfp*. A second PCR product, encompassing the last 64 bp of *spoIIIE* (containing the Shine-Dalgarno sequence of the downstream gene) and the 1 Kb region downstream of *spoIIIE*, was amplified from NCTC8325-4 genomic DNA using primers spoIIIEp5 and spoIIIEp6. The PCR product was digested with XmaI and NcoI and subsequently cloned into pMAD*spoIIIEyfp* generating the plasmid pBCBHV008. The two inserts in pBCBHV008 were sequenced and the plasmid was electroporated into RN4220 and subsequently transduced to strains BCBHV008 and 8325-4*recU*i (selection with erythromycin, chloramphenicol and IPTG), using phage 80α. Integration and excision of pBCBHV008 from the genome was performed as previously described [24] and colonies in which *spoIIIE* had been replaced by the *spoIIIE-yfp* (with the last 64 bp of *spoIIIE* duplicated after the *yfp* gene), were selected by PCR. The BCBHV008 and 8325-4*recU*i strains expressing *spoIIIE-yfp* were named BCBHV017 and BCBRP002, respectively. Functionality of *spoIIIE-yfp* was confirmed by introduction of the fusion protein into a *spoIIIE* null mutant that resulted in complementation of the defective phenotype typical of this strain (data not shown).

### Growth analysis of *S. aureus* strains

Growth of *S. aureus* in liquid culture was analyzed by diluting overnight cultures 1/500 into fresh media, incubating them at 37°C with aeration and following the optical density at 600 nm (OD_600nm_). Strains encoding an inducible *recU* gene and the corresponding control strains were grown overnight in TSB containing chloramphenicol and IPTG. Cells were harvested, washed three times with TSB, and re-inoculated into fresh media supplemented or not with IPTG.

### Western blot analysis

Expression levels of PBP2 were analyzed by western blotting, using a polyclonal anti-PBP2 antibody [31]. A polyclonal anti-FtsZ antibody was used as an internal control. Samples were taken from cultures of BCBHV008 and 8325-4*recU*i supplemented or not with IPTG, grown until an OD_600nm_ 0.5. Cells were broken with glass beads in a Fast Prep FP120 (Thermo Electro Corporation) and unbroken cells were removed by centrifugation. The total protein content of the extracts was quantified by the Bradford method, using bovine serum albumin as a standard (BCA protein assay kit, Pierce). Equal amounts of protein from each sample were loaded onto an 8% SDS-PAGE gel and separated at 120 V. Proteins were then transferred to a Hybond-P Polyvinylidene fluoride (PVDF) membrane (GE Healthcare) using a semidry transfer cell (Bio-Rad). The membranes were cut to separate the region containing PBP2 and FtsZ. Each half of the membrane was blocked with blocking buffer (PBS, 5% milk, 0.5% Tween 20) for 1 hour and incubated with either a polyclonal anti-PBP2 antibody (1/1000 dilution in blocking buffer) or with an anti-FtsZ antibody (1/5000 dilution in blocking buffer) for 16 hours at 4°C. Membranes were washed three times with PBS-T (PBS containing 0.5% Tween 20) and incubated with secondary antibodies (anti-rabbit for PBP2, ECL; anti-sheep for FtsZ, Pierce) diluted 1/100,000 in blocking buffer. The detection was performed using ECL Plus Western blotting detection system (Amersham) according to the manufacturer’s guidelines.

### Fluorescence microscopy

Strains were incubated overnight at 37°C in TSB supplemented with the appropriate antibiotics and IPTG. Cultures were washed three times with fresh TSB and diluted 1/500 in fresh TSB and supplemented with IPTG when required. During exponential phase (O.D_600nm_ 0.5) 1 ml of 8325-4*recUi* culture was taken and incubated with membrane dye Nile Red (5 μg/ml, Invitrogen), DNA dye Hoechst 33342 (1 μg/ml, Invitrogen) and the cell wall dye Van-FL (Invitrogen) mixed in a 1:1(v:v) proportion with non-fluorescent vancomycin (4 μg/ml, Sigma), at room temperature for 5 minutes with shaking. The same protocol was followed for strains BCBHV017 and BCBRP002 but the incubation was performed with the membrane dye FM 5–95 (1 μg/ml, Invitrogen) and with the DNA dye Hoechst 33342 (1 μg/ml, Invitrogen). The cultures were then centrifuged, re-suspended in PBS and 1 μl was placed on a thin layer of 1.2% agarose in PBS. Fluorescence microscopy was performed using a Zeiss Axio Observer.Z1 microscope equipped with a Photometrics CoolSNAP HQ2 camera (Roper Scientific), using Metamorph software (Molecular devices). Analysis of fluorescence images was performed using Metamorph and ImageJ software.

### Determination of mitomycin C minimum inhibitory concentration (MIC)

Determination of the MIC to mitomycin C of 8325-4*recU*i and BCBHV008 strains was performed in liquid medium by micro-dilution. Overnight cultures containing IPTG and chloramphenicol were washed three times with fresh TSB and added at a final cell density of 5×10^5^ CFU/ml to wells containing 2-fold dilutions of mitomycin C in TSB supplemented or not with 0.5 mM IPTG. The 96-well plates were incubated for 24 hours at 37°C and the MIC was recorded as the lowest concentration of mitomycin C that inhibited bacterial growth. All MIC determinations were performed in triplicate.

### UV survival assays

BCBHV008 and 8325-4*recU*i strains were incubated overnight at 37°C with aeration, in TSB supplemented with chloramphenicol and IPTG. These cultures were washed three times with TSB and then diluted 1/500 into fresh TSB, supplemented or not with IPTG and incubated at 37°C until O.D_600nm_ 0.5. Serial dilutions (10^0^ to 10^-6^) were made in TSB and 10 μl of each dilution was spotted on TSA plates containing chloramphenicol and supplemented or not with IPTG. Plates were then irradiated with UV light (Vilber Lourmat, VL-6.LC model, 254 nm) at a dose of 4 J/m^2^ for 0, 10, 20, 30, 40 and 60 seconds and incubated overnight at 37°C in the dark. CFUs were counted and the fraction surviving was determined with reference to an unirradiated control plate.

## Results

### *S. aureus* RecU is required for optimal growth

In order to functionally characterize the RecU homologue in *S. aureus* we deleted the 5’ region of the *recU* gene (encoding the first 165 amino acids) in the background of NCTC8325-4 generating strain 8325-4Δ*recU*. The *recU* gene is encoded upstream of *pbp2,* in the same operon (Figure 1A). This operon contains two promoters, one upstream of *recU* (P1) and the other contained within the *recU* coding sequence (P2) [19]. In order not to affect *pbp2* expression in the *recU* mutant, the last 43 *recU* codons (which contain P2) were not deleted. Growth analysis of the 8325-4Δ*recU* strain indicated that RecU is not essential, but it is required for optimal growth of *S. aureus* since the deletion mutant had a two-fold increase in the doubling time when compared to the parental strain NCTC8325-4 (40 versus 22 minutes, respectively). Given that mutants with very slow growth rates may accumulate suppressor mutations that increase fitness, we generated a *recU* inducible mutant, to be used for further studies. For the construction of this mutant a full copy of *recU* was placed under the control of the IPTG-inducible P_*spac*_ promoter in the ectopic *spa* locus (which encodes for the non-essential Protein A), and subsequently the first 165 codons of *recU* were deleted from the native locus, while in the presence of IPTG (Figure 1A). In order to achieve strong repression of the P_*spac*_ promoter, we introduced the pMGPII plasmid [26], which encodes the *lacI* repressor, generating strain 8325-4*recU*i. Although the two promoters driving expression of *pbp2* are present in this strain, deletion of *recU* decreased the spacing between P1 and P2 promoters. To exclude the possibility that expression of *pbp2* was altered in the 8325-4*recU*i strain, and to ensure that the phenotypes observed in further studies were due only to the absence of RecU and not to low PBP2 levels, we analyzed PBP2 levels in strain 8325-4*recU*i cultured in the presence or absence of IPTG. Figure 1B shows that PBP2 levels are similar in 8325-4*recU*i and the control strain BCBHV008 (where the *spa* gene was replaced by the construct P_*spac*_-MCS-*lacI* and the pMGPII plasmid was introduced), indicating that mutation of *recU* does not affect PBP2 production.

### RecU depletion leads to defects in DNA repair and in chromosome morphology and segregation

**Figure 2:**
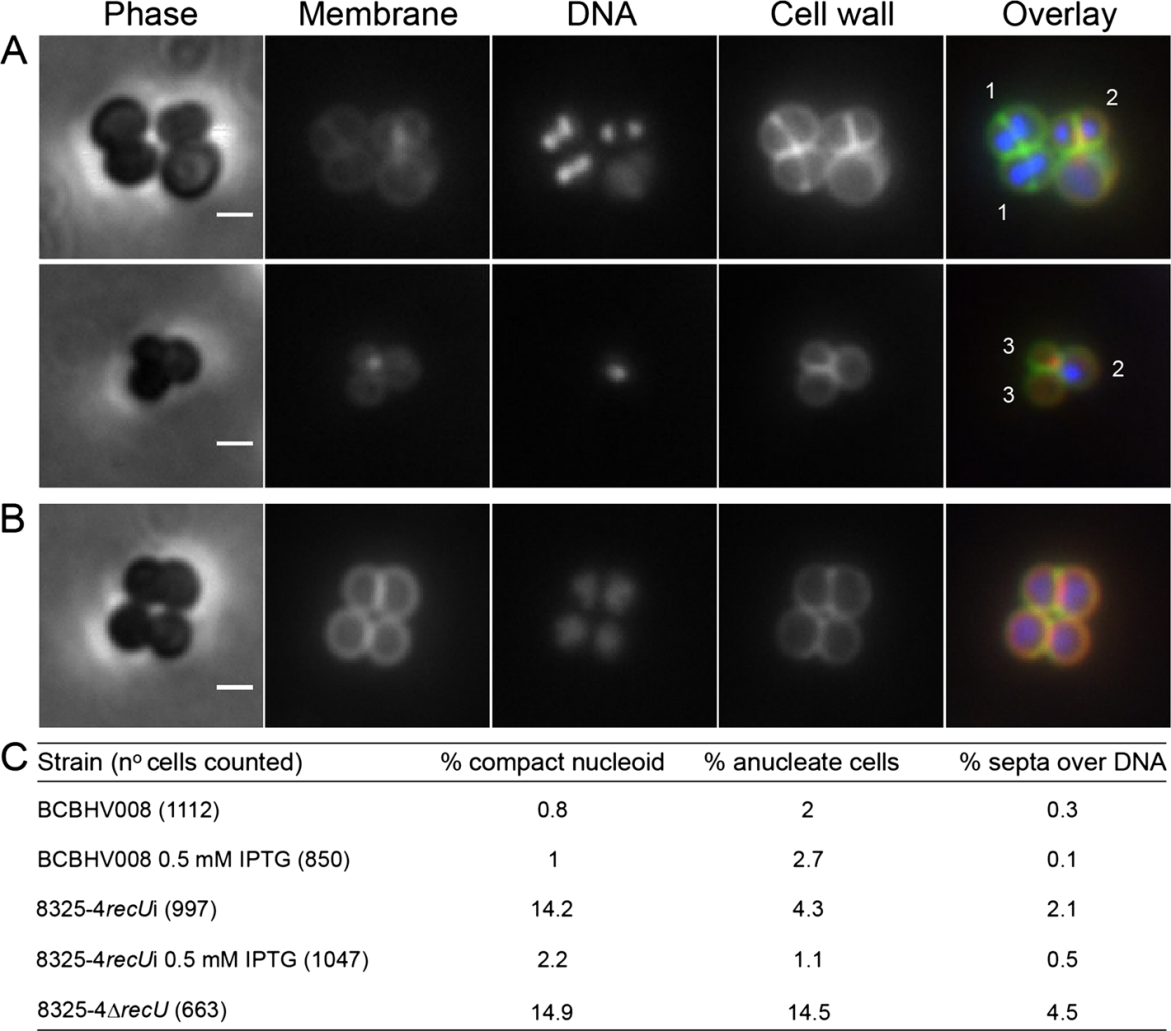
**RecU depletion in *S. aureus *leads to chromosome segregation defects.** The fluorescence microscopy images show cells of *recU* inducible strain 8325-4*recU*i incubated in the absence (**A**) or presence (**B**) of IPTG. Panels from left to right show phase-contrast images, cells stained with membrane dye Nile Red, DNA dye Hoechst 33342, cell wall dye Van-FL and the overlay of the three fluorescence images showing the membrane in red, the DNA in blue and the cell wall in green. The absence of RecU (**A**) led to the formation of cells with septa bisecting the DNA (1), compact nucleoids (2) and anucleate cells (3). Ectopic expression of RecU in 8325-4*recU*i strain, through the addition of IPTG, resulted in the disappearance of the aberrant phenotypes (**B**). Scale bars 1 μm. Panel (**C**) shows a comparison of the phenotypes of control strain BCBHV008; 8325-4*recU* inducible mutant, incubated in the presence or absence of IPTG and 8325-4Δ*recU* mutant.

In order to study the effects of RecU depletion, strain 8325-4*recU*i was incubated in the absence of IPTG for three hours and then observed by fluorescence microscopy (Figure 2). Approximately 14% of the RecU-depleted cells (n = 1046) showed compact nucleoids, while 4% had no DNA (anucleate cells) and 2% presented septa over a compact nucleoid. These phenotypes were shown to be due to the lack of RecU, as they were complemented by ectopic expression of RecU from the *spa* locus (Figure 2B, C). Importantly these phenotypes were also found in cells from the *recU* null mutant strain 8325-4Δ*recU* (Figure 2C) but at a higher frequency. This difference may result from prolonged growth in the absence of RecU in the null mutant or from residual RecU protein present in the inducible strain.

**Figure 3:**
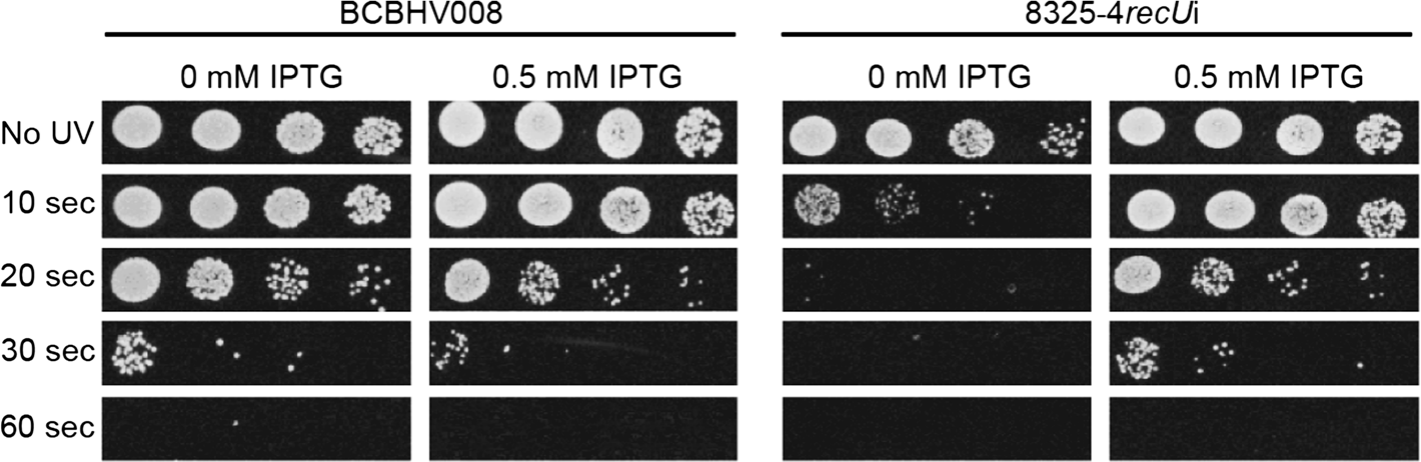
**RecU depletion in 8325-4*recU*i strain leads to increased susceptibility to UV damage.** Cultures of control strain BCBHV008 and *recU* inducible mutant 8325-4*recU*i showing serial dilutions from 10^-2^ (left) to 10^-5^ (right). 10 μl spots were placed on TSA agar, containing or not IPTG, and irradiated with a UV dose of 4 J/m^2^/sec for 0, 10, 20, 30 and 60 seconds. Plates were then incubated overnight and the number of CFU’s was counted.

The presence of anucleate cells can be associated with chromosome segregation defects that result in one sister cell with two chromosomes and another with none. However, they could also arise as a result of DNA degradation caused by DNA guillotining by the septum or due to decreased DNA damage repair. We therefore tested the susceptibility of *recU* mutants to UV light and mitomycin C, both of which cause DNA lesions [32,33]. Depletion of *recU* in the strain 8325-4*recU*i resulted in a 2-fold decrease in mitomycin C MIC (from 0.8 to 0.4 ng/ml), compared to the same strain grown in the presence of IPTG or to the control strain BCBHV008. Importantly, addition of IPTG recovered the MIC to wild-type levels. Similar results were obtained for the null mutant strain 8325-4Δ*recU* which had a 6-fold decrease in the mitomycin MIC compared to the parental strain. RecU depletion also caused *S. aureus* to become more sensitive to UV damage, since 10 sec of exposure time to UV light were sufficient to kill approximately 99% of the 8325-4*recU*i cells grown in the absence of ITPG but had no significant effect on BCBHV008 cells or 8325-4*recU*i cells grown in the presence of the inducer, which required 20 sec of UV exposures for similar decrease in cell viability (Figure 3). Taken together, these results indicate that RecU is required for DNA damage repair in *S. aureus* and that its ectopic expression from the *spa* locus was sufficient to fully recover UV and mitomycin C resistance to wild type levels.

### Absence of RecU leads to increased recruitment of the SpoIIIE DNA pump to the division septum

**Figure 4:**
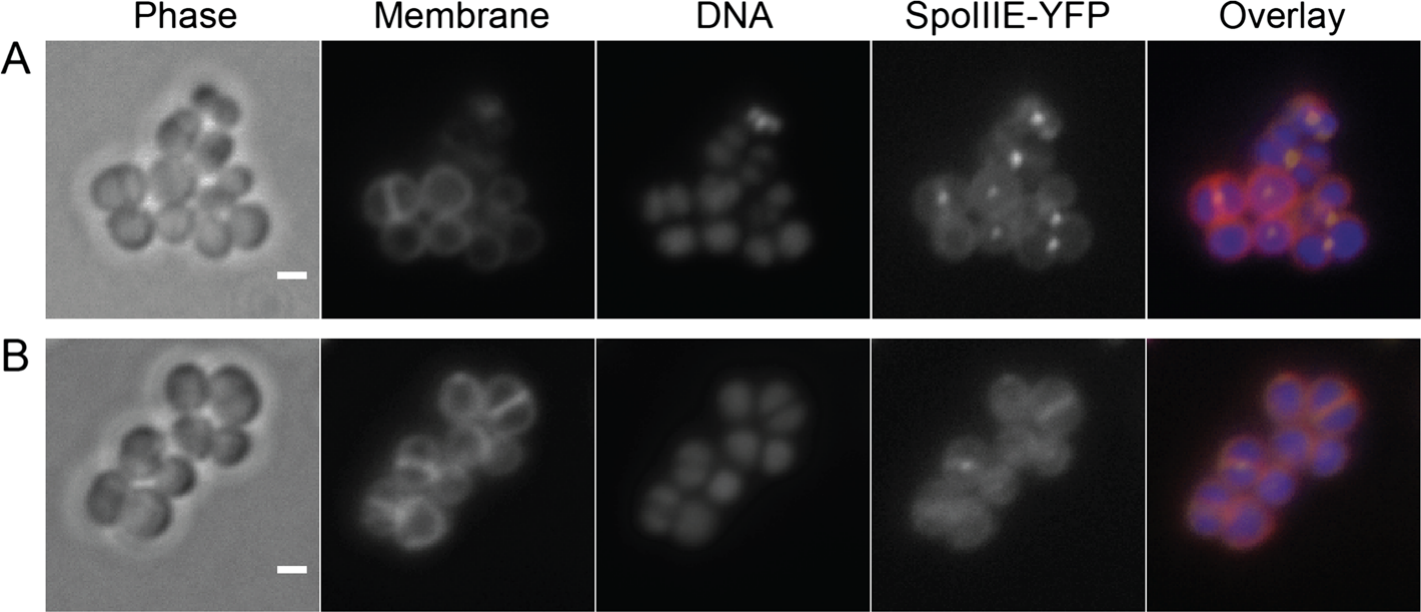
**RecU-depleted cells show increased frequency of SpoIIIE-YFP foci.** The figure shows SpoIIIE-YFP localization in *recU* inducible strain BCBRP002 incubated in the absence (**A**) or presence (**B**) of IPTG. SpoIIIE-YFP foci are present in 44% of BCBRP002 RecU-depleted cells in comparison with 10% of the cells of the same strain when expressing RecU. Panels from left to right show phase-contrast image, membrane labeled with FM 5–95, DNA stained with Hoechst 33342, SpoIIIE-YFP localization, and the overlay of the three fluorescence images showing the membrane in red, DNA in blue and SpoIIIE-YFP in yellow. Scale bars 1 μm.

SpoIIIE is a DNA pump crucial for moving DNA into the forespore of *B. subtilis* during sporulation [34]. During vegetative growth of *B. subtilis* this protein plays an important backup role when the chromosome fails to segregate prior to septum formation [35-37]. The presence of SpoIIIE foci localized near the center of the septum in a small fraction (~6%) of vegetatively growing *B. subtilis* cells is thought to reflect its role in post-septational chromosome partioning [38]. Therefore, we have used SpoIIIE recruitment to the septum as a marker for the requirement to resolve chromosome segregation defects. For that purpose we fused SpoIIIE to the yellow fluorescent protein YFP and expressed this fusion protein in the 8325-4*recU*i background, generating the strain BCBRP002 (Figure 4). SpoIIIE-YFP foci were present in 10% (n = 580) of the cells cultured in the presence of inducer. However, when the same strain was cultured in the absence of IPTG, the number of cells with SpoIIIE-YFP foci increased to 44% (n = 536). In a control experiment, addition of IPTG did not change the fraction of cells exhibiting SpoIIIE foci in the control strain BCBHV017, a strain identical to BCBRP002 but lacking the *recU* mutations (data not shown). These results suggest that RecU is required for correct segregation of the *S. aureus* chromosome as its absence increases the need for SpoIIIE-mediated post-septational chromosome partitioning.

## Discussion

The role of RecU in homologous recombination and in DNA repair has been well studied in a small number of organisms [39-41]. However DSB repair mechanisms studied in one bacterial species cannot be directly extrapolated to other species since the phenotypes that arise from the same mutations in different bacteria are not always the same [42]. Furthermore, homologous recombination has an important role in the evolution of antibiotic resistance and acquisition of virulence determinants [15,16], emphasizing the relevance of studying this mechanism in pathogenic bacteria.

We have now studied the role of RecU in the clinical pathogen *S. aureus* and found that the major phenotypes observed in RecU depleted *S. aureus* cells were compatible with defects in chromosome segregation and DNA repair. These phenotypes include: (i) The presence of anucleate cells, which can result from deficient chromosome partioning causing one of the daughter cells to inherit the two copies of the genome and the other none. Alternatively, anucleate cells can arise from DNA degradation resulting from DNA breaks due to chromosome guillotining by septum placement over the nucleoid [12,23] or from DNA damage that is not repaired [43]. (ii) Compaction of the nucleoid, a phenotype that has already been observed in *B. subtilis* and *E. coli* under DNA damaging conditions, such as UV irradiation. Interestingly, these observations led to the suggestion that a dramatic alteration of nucleoid morphology may be part of an active mechanism to protect the cell’s genome when DNA repair is required [43-45], a mechanism which we now suggest also occurs in *S. aureus.* (iii) Increased sensitivity to UV irradiation and mitomycin C, a phenotype in agreement with a role of RecU in DNA damage repair. (iv) Increased recruitment of the DNA translocase SpoIIIE. In *B. subtilis,* RecU has been shown to bias homologous recombination towards non-crossover products [7,11], decreasing the formation of chromosome dimers that would not be properly segregated into the daughter cells [46-48]. When present, chromosome dimers can be resolved by dedicated recombinases in a process that requires the presence of at least one of the two DNA translocases, SpoIIIE or SftA [49]. Furthermore, the presence of septal SpoIIIE foci was proposed to be associated with its role in post-septational chromosome partitioning [38]. Therefore, the fact that approximately half of the *S. aureus* cells grown in the absence of RecU had SpoIIIE-YFP foci (compared to 10% of the cells grown in its presence), suggests that RecU has a major role in chromosome segregation, maybe through biasing recombination towards non-crossover products. (v) The presence of septa placed over the DNA, a phenotype that could be caused by segregation defects or, alternatively, by the lack of a cell division checkpoint required to prevent septum formation over the DNA (see below). Together, the phenotypes observed for RecU depleted cells strongly point to an important role of this protein in DNA repair and chromosome segregation, in agreement with what would be expected for a Holliday junction resolvase.

In the course of *S. aureus* cell division, the synthesis of cell wall occurs at the septum, which progressively closes to originate the two daughter cells. During this process the chromosome is replicated and the two resulting DNA molecules are segregated. Tight coordination between chromosome segregation (which requires RecU) and septum synthesis (which requires PBP2, encoded in the same operon as RecU), two biosynthetically unrelated events, is therefore essential for proper division, to ensure that the septum does not form over the nucleoid, which would result in DNA damage. Given that the genetic organization of the *recU-pbp2* operon is maintained in other gram-positive bacteria [19,21,22], we hypothesized that co-regulation of the expression of these two proteins could be central for the coordination of cell division events. We have abolished this co-regulation (but maintained the presence of RecU in the cell) in strain 8325-4*recU*i by placing an inducible copy of *recU* in the distant *spa* locus, under the control of the P_*spac*_ promoter and deleting the native gene from the *recU-pbp2* operon. When this mutant is incubated with IPTG, RecU is produced from the ectopic *spa* locus while PBP2 is expressed from its native locus, under the control of its native promoters. If *recU/pbp2* co-regulation constituted a checkpoint for cell division, we should detect a subpopulation of cells with cell division defects when the 8325-4*recU*i strain was incubated with IPTG. This is not what we have observed, since ectopic expression of *recU* led to a reversal of the phenotypes observed in the absence of RecU, namely the presence of anucleate cells and cells with septa over DNA (Figure 2A-C). This indicates that although RecU may have a role in preventing chromosome trapping by the septum, co-regulation of *recU* and *pbp2* expression from the same operon is not required during cell division.

## Conclusions

We have shown that lack of *S. aureus* RecU protein has important consequences in the cells, doubling the duplication time, increasing the susceptibility to DNA damage and leading to the appearance of a large population of cells with compact nucleoids, lacking a nucleoid or with septa placed over the chromosome. This shows that the role of RecU in chromosome segregation and DNA repair is crucial for normal growth of *S. aureus* cells. RecU is encoded in the same operon as the cell wall synthesis protein PBP2 and consequently the two proteins are overexpressed under certain conditions, such as in the presence of cell wall targeting antibiotics [50]. We have shown that this genetic organization is not required for correct cell division in rich medium, but it remains to be determined if it becomes advantageous under other, more clinically relevant, conditions.

## Competing interests

The authors declare that they have no competing interests.

## Authors’ contributions

ARP, PR and MGP designed research, analyzed data and wrote the paper, HV contributed with new genetic constructs, ARP performed research. All authors read and approved the final manuscript.
