# Erratum to: The Holliday junction resolvase RecU is required for chromosome segregation and DNA damage repair in *Staphylococcus aureus*

**DOI:** 10.1186/s12866-017-1026-2

**Published:** 2017-05-22

**Authors:** Ana R. Pereira, Patricia Reed, Helena Veiga, Mariana G. Pinho

**Affiliations:** Laboratory of Bacterial Cell Biology, Instituto de Tecnologia Química e Biológica, Universidade Nova de Lisboa, Av. da República, 2780-157 Oeiras, Portugal

## Erratum

Following the publication of our article [1] in *BMC Microbiology*, it was brought to our attention that there was an error in Fig. 3: the panels showing BCBHV008 and 8325-4*recU*i supplemented with IPTG (2nd and 4th panels respectively) were identical. After checking the original files, we concluded that the images used to create the two panels came from the same files (corresponding to 8325-4*recU*i plus IPTG) and the original data for the control strain BCBHV008 plus IPTG had not been saved. Therefore we repeated the experiment described in Fig. 3. We achieved the same result, i.e. that BCBHV008 susceptibility to UV is not affected by the presence of IPTG, as expected given that this control strain does not encode any gene under the control of the Pspac IPTG inducible promoter. Hence, Fig. 3 should be replaced by the correct version below. This correction does not affect any of the results or the conclusions.

**Fig. 3:**
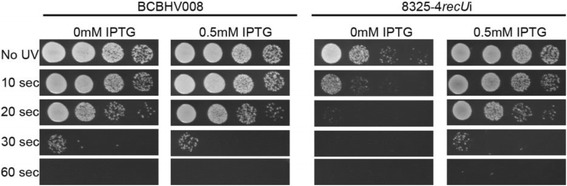
RecU depletion in 8325-4*recU*i strain leads to increased susceptibility to UV damage. Cultures of control strain BCBHV008 and *recU* inducible mutant 8325-4*recU*i showing serial dilutions from 10^−2^ (*left*) to 10^−5^ (*right*). 10 μl spots were placed on TSA agar, containing or not IPTG, and irradiated with a UV dose of 4 J/m^2^/s for 0, 10, 20, 30 and 60 s. Plates were then incubated overnight at 37 °C
